# A combination of carbonates and Opuntia ficus-indica extract protects esophageal cells against simulated acidic and non-acidic reflux in vitro

**DOI:** 10.1038/s41598-024-74047-7

**Published:** 2024-09-27

**Authors:** Martin D. Lehner, Ulrike Scheyhing, Jens Elsässer

**Affiliations:** grid.476242.1 0000 0004 0390 2958 Dr. Willmar Schwabe GmbH & Co. KG, Willmar-Schwabe-Str. 4, 76227 Karlsruhe, Germany

**Keywords:** Antacids, Calcium carbonate, Magnesium carbonate, Prickly pear, Bile acid, Heartburn, Gastrointestinal models, Gastro-oesophageal reflux disease, Preclinical research, Drug development

## Abstract

Buffering of stomach acid by antacids is a well-established symptomatic therapy for heartburn. In addition, preparations from prickly pear (*Opuntia ficus-indica*) have been shown to reduce tissue damage in experimental gastritis models and to attenuate gastrointestinal discomfort in patients. Both active principles have been included in a fixed-combination product for symptomatic treatment of heartburn containing carbonate antacids (CaCO3 and MgCO3) and an extract from *Opuntia ficus-indica* cladodes. The aim of the study was to characterize the acid neutralization and esophageal cell protective activities of the product and its individual active ingredients in a set of in vitro assays. Acid neutralization was assessed in a simulated stomach model. Protective activity of individual constituents and in combination was analyzed in an esophageal cell line (COLO-680 N) exposed to low pH and deoxycholic acid to simulate acidic and non-acidic reflux challenge. The combination product protected cells against low pH mediated cytotoxicity via acid neutralization by carbonates. Opuntia extract itself and the combination product attenuated bile acid-induced cell irritation as measured by reduced release of proinflammatory interleukin-6 and -8. In conclusion, addition of Opuntia extract to a mineral antacid provides dual protection against acidic and non-acidic simulated reflux challenge.

**Supplementary Information:**

The online version contains supplementary material available at 10.1038/s41598-024-74047-7.

## Introduction

Gastroesophageal reflux disease (GERD) is a common ailment and one of the most important reasons for gastric discomfort with a high world-wide prevalence. The chronic and relapsing occurrence of GERD is associated with a significant reduction in health-related quality of life characterized by disturbed sleep, reduced vitality, generalized body pain, an impaired sex life and anxiety about the underlying cause of the symptoms^1,2^.

Mechanistically, the reflux of stomach content into the esophagus is associated with disruption of mucosal barrier integrity and sensitization of nociceptive chemoreceptors, leading to the sensation of acid regurgitation and heartburn^3,4^. Upon repeated exposure, secondary inflammatory responses of the epithelium contribute to sensitization via impairment of epithelial cell tight junction expression or modulation of TRP channels activity on nociceptors^4^.

Treatments of heartburn involve life-style changes such as weight reduction, avoidance of alcohol, tobacco and meals rich in fat and salt^2^.

The mainstay of symptomatic treatment consists in the control of stomach acidity. This can be accomplished by suppression of acid production by proton pump inhibitors (PPIs) or histamine (H2) receptor antagonists^5^. One of the most commonly used self-medication therapies is based on neutralization of gastric acid by so called antacids, i.e. mineral salts containing pH increasing/neutralizing anions accompanied by aluminum-, calcium-, magnesium- or sodium cations. Unlike PPIs or histamine (H2) receptor antagonists, antacids do not inhibit the production of hydrochloric acid by the parietal cells in the stomach but directly reduce acidity of the stomach content by neutralization of hydrochloric acid present in the stomach. Therapies with different antacids have been shown to lead to a rapid, albeit transitory neutralization of stomach acidity and symptomatic improvements^5,6^.

Despite the high relevance of stomach acidity for GERD symptoms, the latter persist despite profound suppression of stomach acidity by PPI therapy in a relevant number of patients^7^. This suggests that not only cell irritation mediated by direct acid and pepsin (which is acid-dependent in its activity) but also exposure to other gastric content constituents contributes to reflux symptoms. Hence, broad-acting treatment approaches based on the creation of physical barriers to prevent direct contact of stomach content with the esophageal mucosal cells could provide an acid-independent additional protective activity.

Cladodes from *Opuntia ficus-indica* (L.) Mill. [Cactaceae] (prickly pear or nopal cactus) are traditionally used as an ulcer treatment in Sicilian folk medicine^8^. Non-clinical studies have shown protective activity of different *Opuntia* cladode-based preparations in animal models of experimentally induced gastritis ^8–11^. In addition, the therapeutic efficacy of orally administered *Opuntia* cladode based extracts has been demonstrated with two products containing *Opuntia* extract as one of the active principles in clinical studies in patients suffering from GERD and gastrointestinal discomfort^12,13^.

Fractionation studies suggested that the protective activity in some of the studies was mediated at least in part by high-molecular polysaccharides^9,11^.

Upon contact with water, these high molecular weight compounds produce colloidal solutions of high viscosity^14^. This activity could contribute to the mucoadhesive effects of *Opuntia* extract shown by in vitro studies with colonic CaCo-2 cells^15^.

Based on these preclinical data, it seems plausible that the mechanism of the reported beneficial effects of Opuntia extracts is the formation of viscous films on mucosal cells. These films could act as a physical protective barrier and thereby reduce access of noxious stimuli to the epithelial surface.

Due to the reported activity of *Opuntia ficus-indica*-based preparations, a new fixed combination product has recently been developed for symptomatic treatment of heartburn and recurrent acid-related gastric discomfort. It contains mineral salt-based antacids (CaCO_3_ & MgCO_3_) supplemented with a polysaccharide-rich extract containing mucoprotective substances from *Opuntia ficus-indica* cladodes, aimed to confer additional physical protection to the esophageal mucosa.

In the present study we assessed the activity profile of the individual active constituents both separately and in combination in form of the final product in terms of pH neutralization and protection of esophageal cells against different reflux-simulating challenges in vitro. We show that the addition of *Opuntia* extract adds protective activity against simulated non-acidic, bile acid-induced reflux irritation and thus complements the well-established activity of carbonate-based antacids against acid-induced irritation.

## Results

### Assessment of acid neutralization capacity

**Fig. 1:**
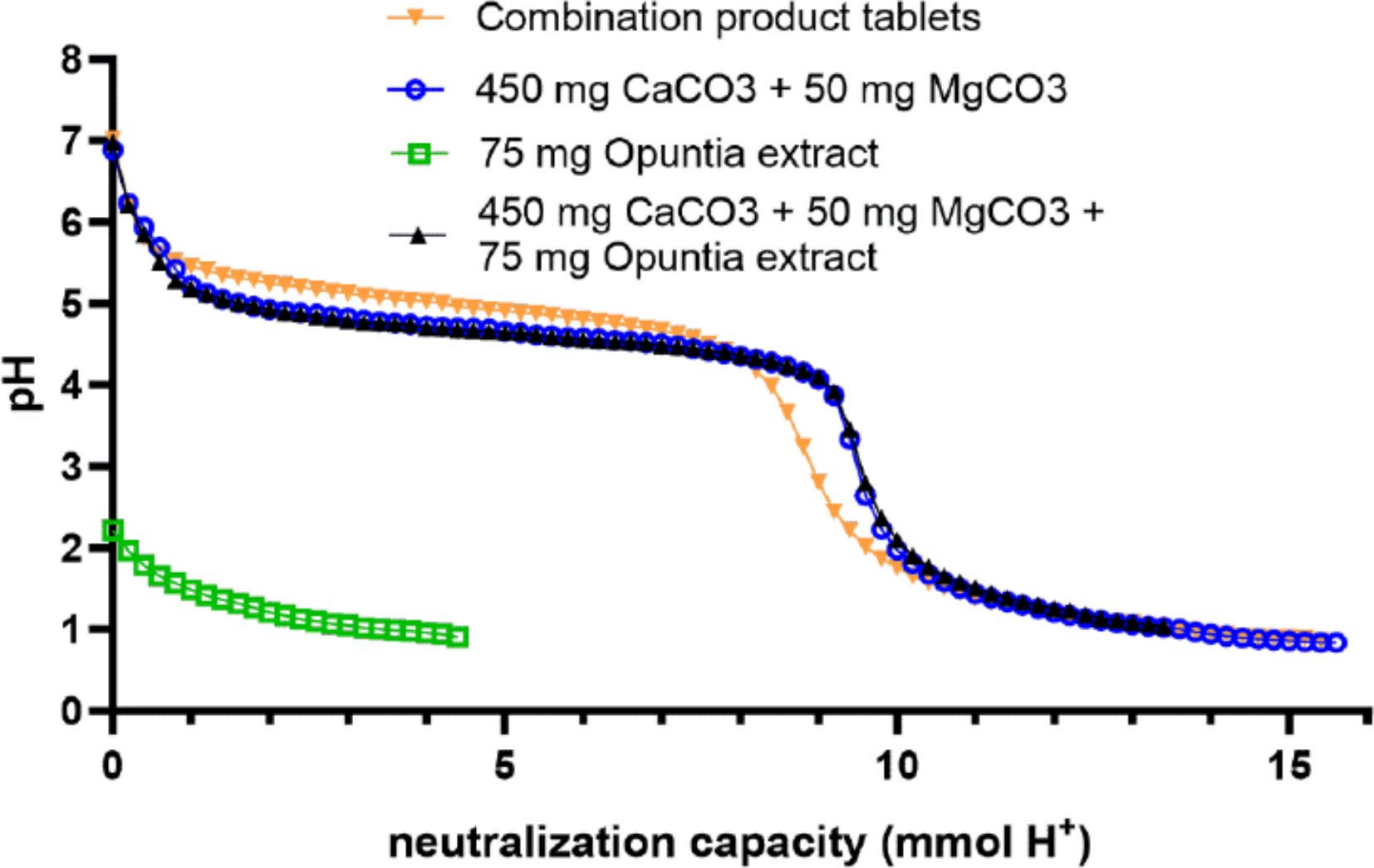
Acid neutralization capacity. One ground combination product tablet or the corresponding amounts of active constituents were added to 40 mL of artificial gastric juice at pH 2.0. Neutralization capacity was quantified by pH determination during constant addition of 1 M HCl solution.

The buffer capacity of the individual components and the combination is presented as a plot of pH over mmol H^+^ (Fig. 1).

The ground combination product tablets displayed a buffer capacity of approximately 9 mmol HCl and maintained the pH of the artificial stomach juice with constant addition of HCl between pH 4.5–5.5 during its buffer capacity. The buffer capacity of the combination product tablets was exclusively contributed by the carbonates (buffer capacity of 9.5 mmol HCl) and was not affected by addition of *Opuntia* extract (buffer capacity of 9.5 HCl after addition of Opuntia to carbonates). *Opuntia* extract itself did not show any relevant effects on the course of the pH.

### Determination of pH neutralization kinetics

**Fig. 2:**
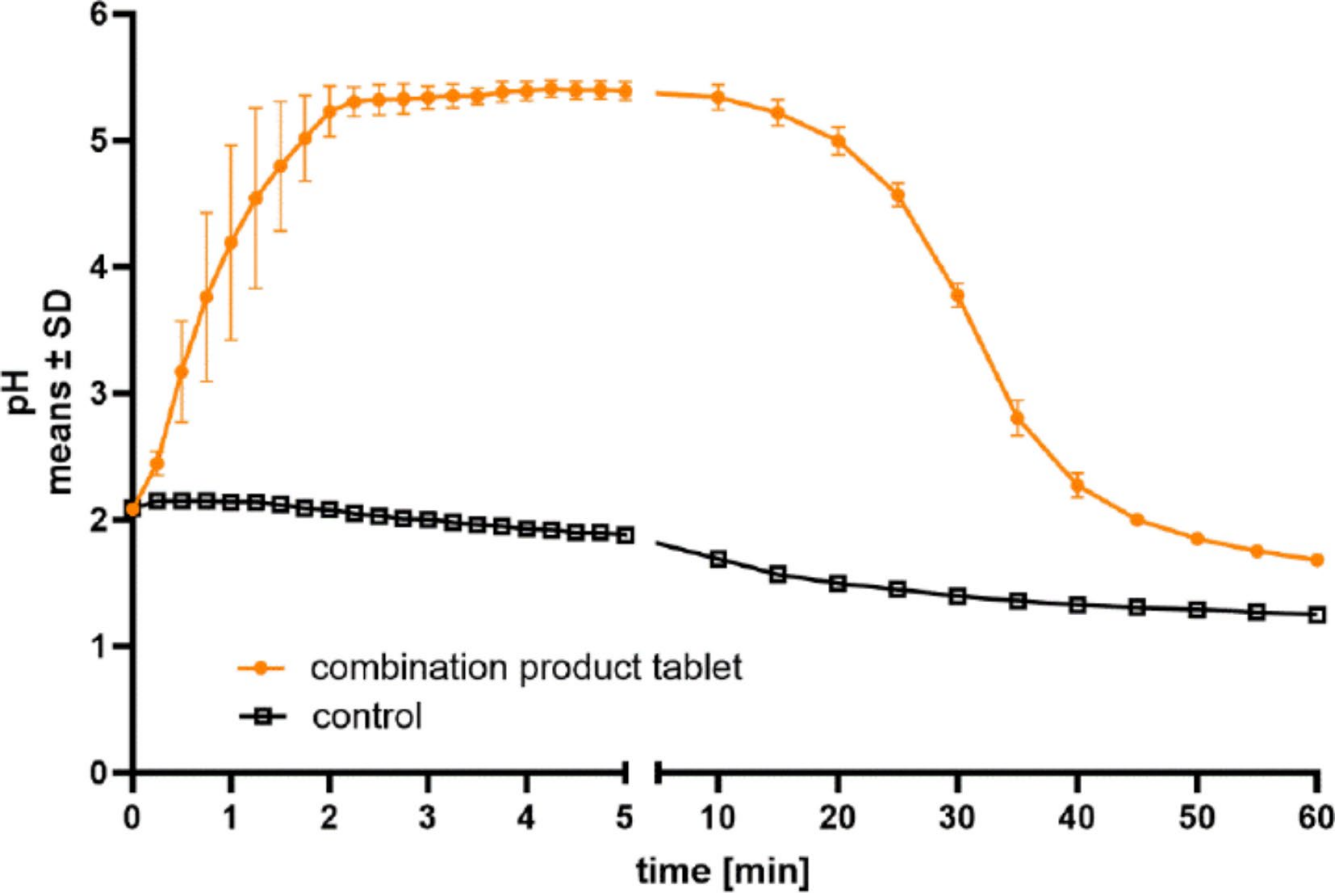
Kinetics of pH neutralization in simulated stomach model. 5 mL of artificial saliva or a suspension of a ground combination product tablet in saliva was added to 100 mL of artificial gastric juice in a simulated stomach model with constant addition of HCl and removal of gastric juice. Addition of a single tablet rapidly increased pH of gastric juice which was maintained at pH > 4.0 for approximately 28 min. Mean values ± SD of pH measurements of 3 independently conducted studies are shown.

As a surrogate marker for onset of activity we analyzed the kinetics of pH neutralization of a stirred artificial stomach juice after addition of the combination product dissolved in artificial saliva to simulate the chewing process (Fig. 2).

Upon addition of 5 mL artificial saliva (control) to the stomach model, no relevant changes of pH were observed (increase from pH 2.0 to 2.15). Due to the constant addition of 3 mL/min of 0.1 M HCl and removal of 1.5 mL/min of stomach juice the pH continuously decreased to 1.25 at 60 min. In contrast, the addition of a single combination product tablet suspended in 5 mL artificial saliva led to a rapid increase to pH 5.3 within 3 min, indicating a rapid dissolution of the suspension and neutralization of acid by the carbonate constituents. In our setup with constant addition of HCl and removal of stomach content, the pH was maintained above pH 4 for approximately 28 min and pH returned to the baseline value of pH 2 by 45 min.

### Protective effect of the combination product against low pH-induced cytotoxicity is mediated by carbonates

**Fig. 3:**
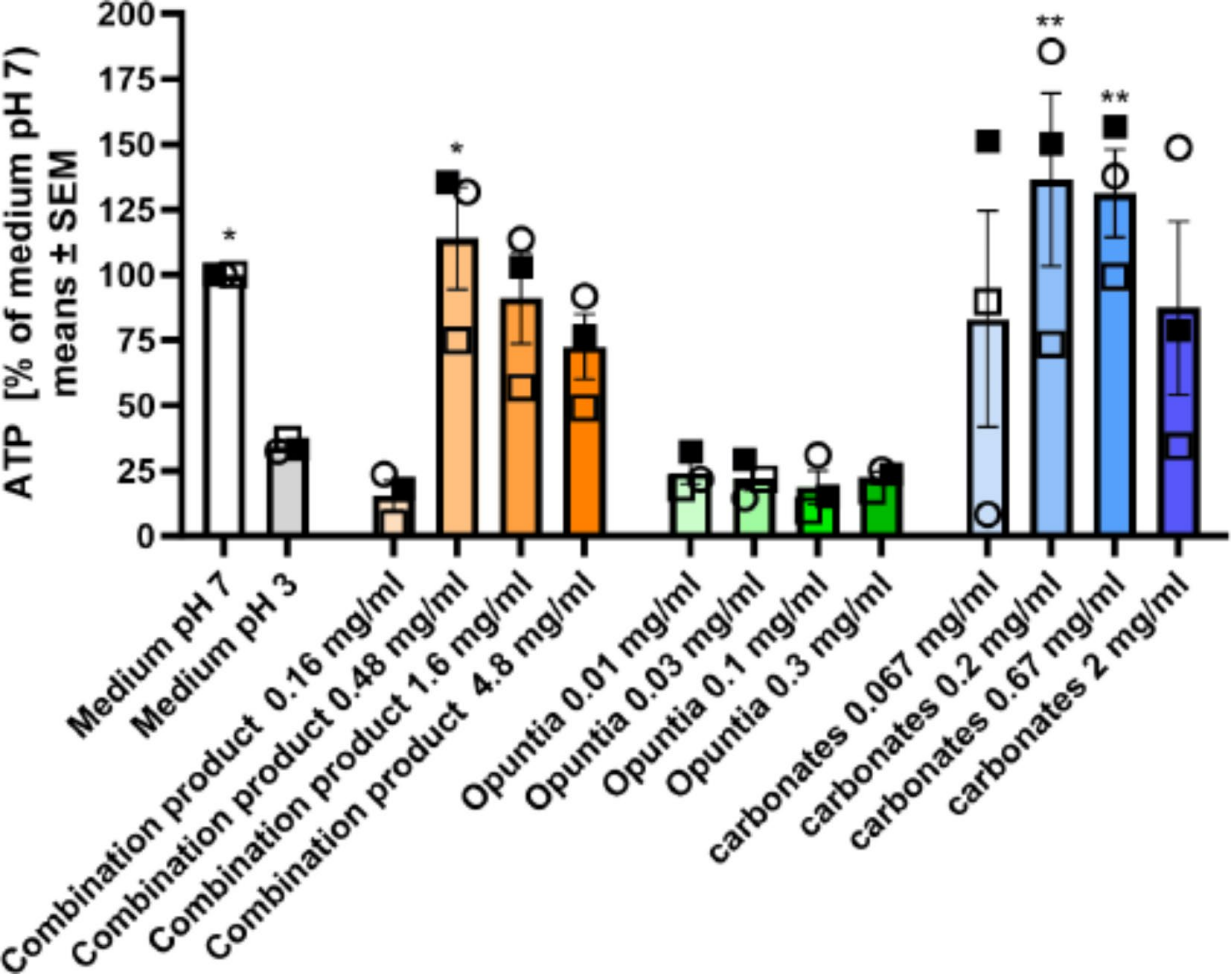
Protective effect of the combination product and carbonates against cytotoxicity induced by low pH. Pretreatment of COLO-680 N with ground combination product tablets and corresponding amount of carbonates, but not of Opuntia extract attenuated acid-mediated cytotoxicity (3 h incubation at pH 3.0). Means ± SEM of mean values of 3 independently conducted studies. *p < 0.05, **p < 0.01, assessed by two-way ANOVA, followed by Dunnett´s test vs. medium pH 3.0.

In our experiments we established a 3 h exposure time at pH 3.0 as a suitable condition to assess cytotoxicity induced by low pH in COLO-680 N cells. This condition resulted in a reduction of cell viability as assessed by ATP measurement to approximately 34% of control cells (Fig. 3).

Treatment of cells with the combination product at concentrations of 0.48–4.8 mg/mL largely normalized cell viability to levels similar to control conditions. At 0.16 mg/mL no effect was observed, indicating that the tablet concentration was too low. The apparent reduction in the protective effect at higher concentrations of ≥ 4.8 mg/mL suggests negative effects of the tablet on viability of cells independent of pH, probably via changes in osmolality of the medium that affected viability of cells upon prolonged exposure. When we assessed the individual components at the concentrations corresponding to its content in the ground tablet, carbonates but not *Opuntia* extract produced a significant protective effect on viability, comparable to the effect of the combination product. This indicates that the protective effect of the tablet was primarily due to neutralization of the acidic pH by carbonates.

### Kinetics of bile acid-induced inflammatory cytokine induction

**Fig. 4:**
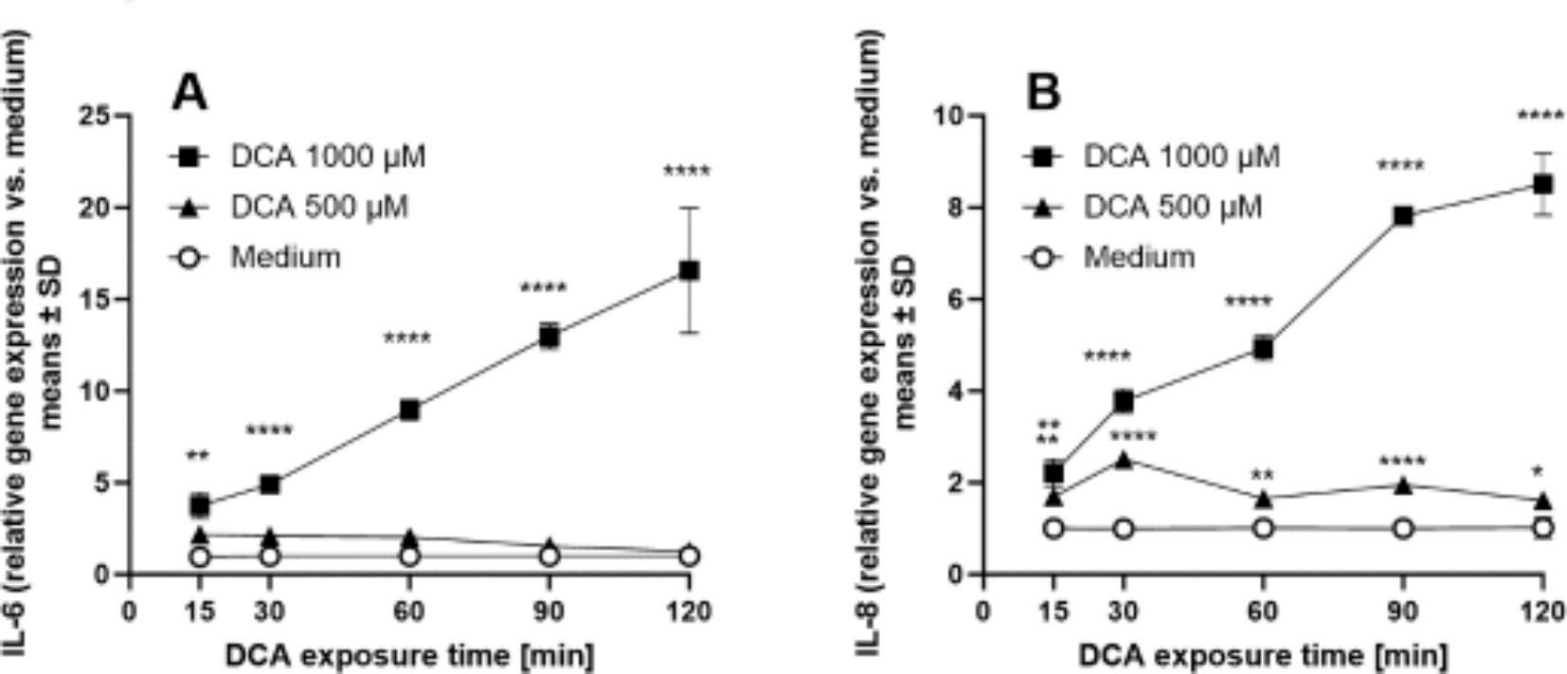
Kinetics of inflammatory cytokine induction by DCA. Short-term exposure of COLO-680 N to DCA 1000 µM prior to wash-out leads to a concentration- and exposure time-dependent induction of IL-6 mRNA (**A**) and IL-8 mRNA (**B**) (mRNA isolated at 4 h after wash-out). Means ± SD of 3 replicates per condition. *p < 0.05, **p < 0.01, **** *p* < 0.0001 assessed by two-way ANOVA, followed by Dunnett’s test vs. medium.

To enable an assessment of protective treatment effects independent of pH neutralization by carbonates, we first established conditions of non-acidic reflux-mediated cell irritation induced by the bile acid DCA. In time course studies we determined optimal concentrations and the necessary minimal exposure time to detect cell irritation measured as gene induction of *IL-6* and *IL-8* in COLO-680 N cells. Addition of DCA at 1000 µM for different exposure times prior to wash-out led to a time-dependent increase in *IL-6* and *IL-8* mRNA levels, analyzed at 4 h after wash-out of DCA. Significant increases were observed already with a 15 min exposure time (Fig. 4A & B).

A lower concentration of DCA of 500 µM did not result in robust induction of either cytokine mRNA in this wash-out setting. Based on these results, we selected a DCA concentration of 1000 µM and an exposure time of 30 min for subsequent intervention studies.

### Opuntia extract protects against DCA-induced cell irritation

**Fig. 5:**
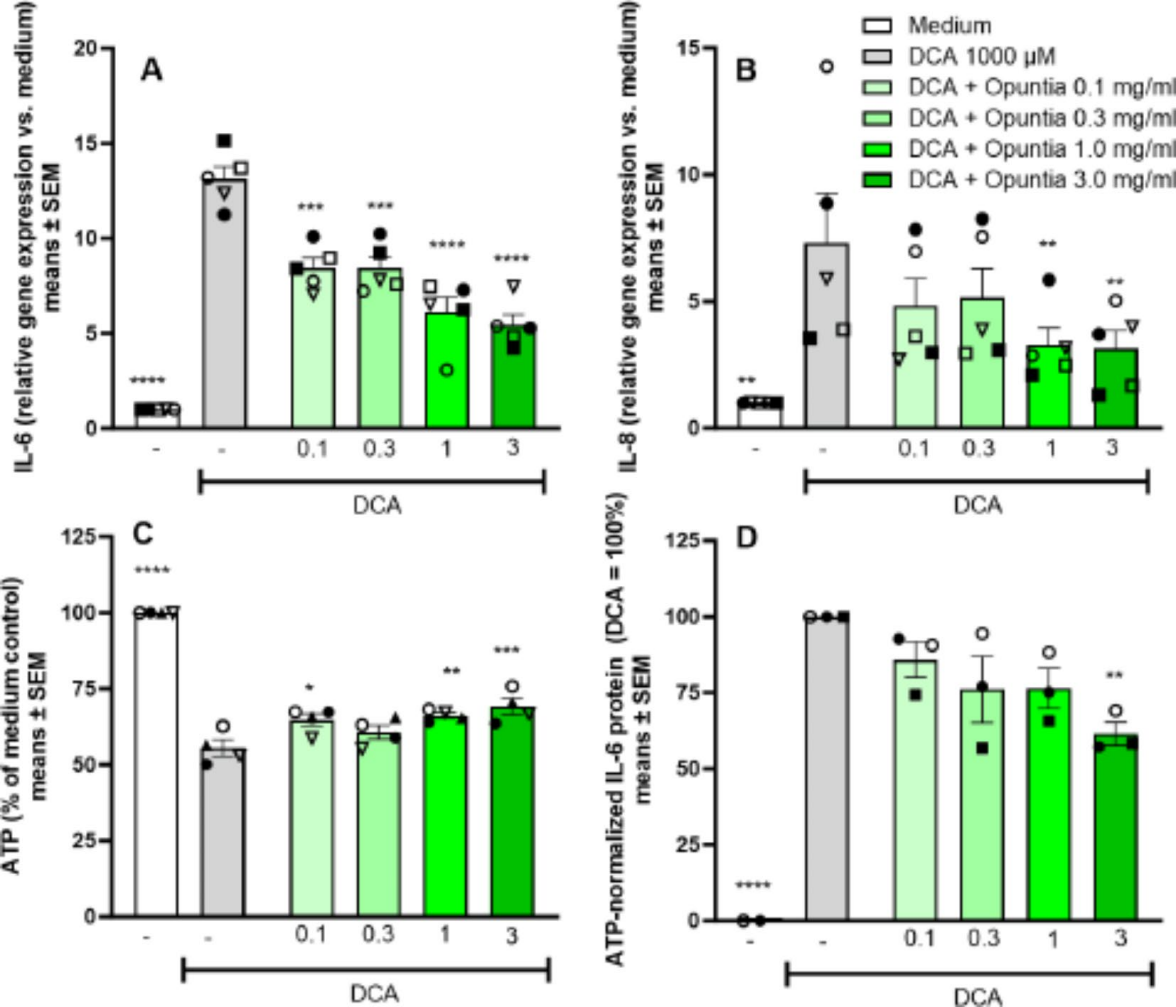
Opuntia extract attenuates DCA-induced proinflammatory gene induction, cytotoxicity and IL-6 protein production. Pretreatment of COLO-680 N with Opuntia extract prior to a 30 min challenge with DCA attenuated the DCA-induced increase in IL-6 mRNA (**A**) and IL-8 mRNA (**B**) (mRNA isolated at 4 h after wash-out). At 24 h, Opuntia extract attenuated DCA-mediated cytotoxicity (**C**) and reduced release of IL-6 protein in supernatant (**D**). Means ± SEM of mean values of 3–5 independently conducted studies. **p < 0.01, **p < 0.01, ***p < 0.001, ****p < 0.0001 assessed by two-way ANOVA, followed by Dunnett´s test vs. DCA.

Treatment of COLO-680 N cells with *Opuntia* extract at concentrations of 0.1-3 mg/mL prior to a 30 min exposure to DCA produced a concentration-dependent attenuation of cell irritation as assessed by gene induction levels of *IL-6* and *IL-8* (Fig. 5A & B).

Maximal effects were found at the highest test concentration of 3 mg/mL. At this concentration gene induction was reduced from 13.1 ± 1.5 (DCA means ± SD) to 5.5 ± 1.3 (DCA + *Opuntia*) and 7.3 ± 4.4 (DCA) to 3.1 ± 1.6 (DCA + *Opuntia*), for *IL-6* and *IL-8*, respectively.

Effects on cell viability and IL-6 protein in supernatant were analyzed in separate plates at 24 h after wash-out of DCA. DCA produced a significant reduction in ATP content, indicating relevant cytotoxic effects at the analyzed 24 h time point. Addition of *Opuntia* extract led to a modest and concentration dependent increase in ATP levels (Fig. 5C), suggesting an attenuation of DCA-induced cytotoxicity. In supernatants we additionally analyzed IL-6 protein levels at 24 h. Exposure to DCA produced an increase in IL-6 protein concentration in the supernatant. Absolute IL-6 levels strongly varied between the three assays (medium controls: 1148, 1955, 579 ng/mL; DCA controls: 2488, 10459, 8415 ng/mL) and treatments affected viability and thus the number of IL-6 producing cells. Therefore, a normalization of IL-6 to ATP levels and the respective DCA control in each assay plate was conducted to combine the individual study results in a single analysis. In this analysis, treatment with *Opuntia* extract led to a modest and concentration-dependent reduction in IL-6 protein release with significant reduction to 61.5 ± 6.6% of DCA controls by the highest Opuntia extract concentration of 3 mg/mL (Fig. 5D).

### Combination product protects against DCA induced gene induction

**Fig. 6:**
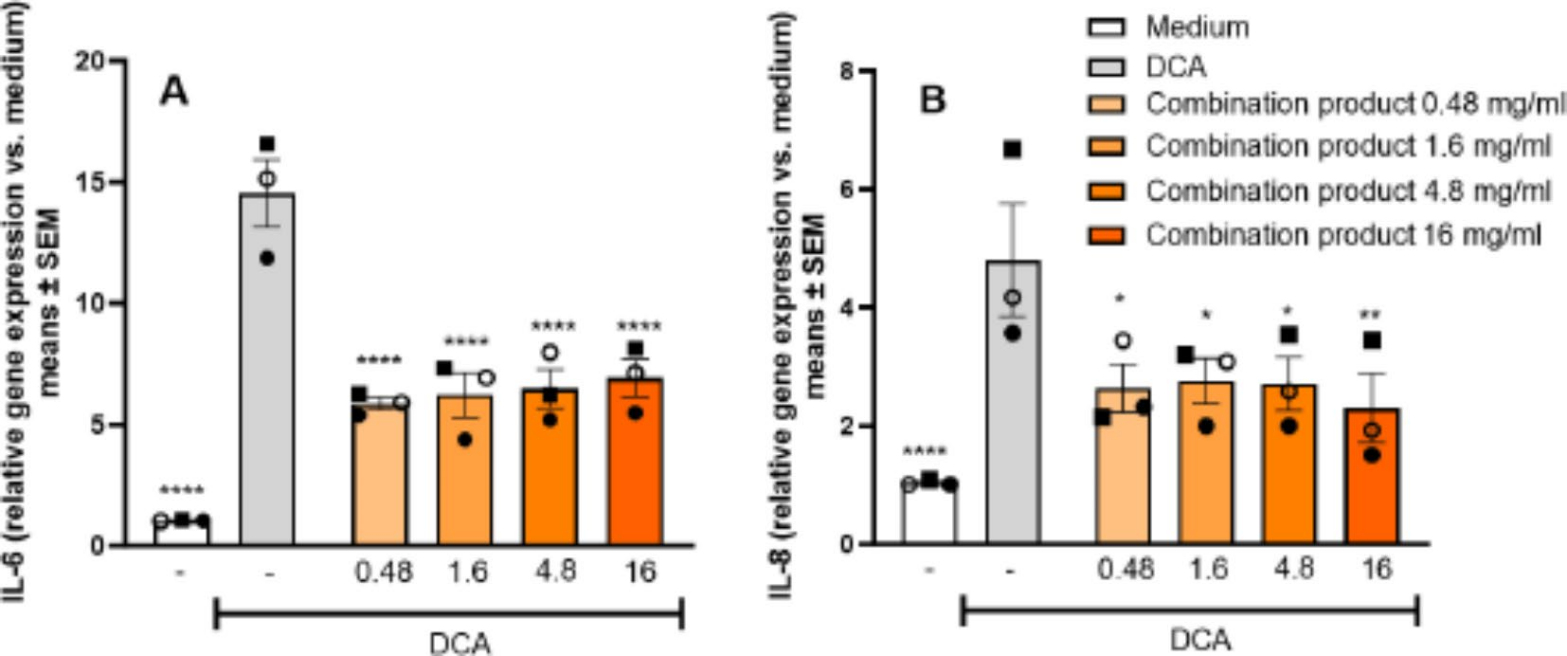
Combination product attenuates DCA-induced proinflammatory gene induction. Treatment of COLO-680 N with ground combination product tablets prior to 30 min challenge with DCA attenuated the DCA-induced increase in IL-6 mRNA (**A**) and IL-8 mRNA (**B**) (mRNA isolated at 4 h after wash-out). Means ± SD of mean values of 3 independently conducted studies. *p < 0.05, ***p < 0.001, ****p < 0.0001 assessed by two-way ANOVA, followed by Dunnett´s test vs. DCA.

Treatment of COLO-680 N cells with ground combination product tablets at concentration of 0.48-16 mg/mL prior to a 30 min exposure to DCA attenuated cell irritation as assessed by gene induction of *IL-6* and *IL-8* with maximal reductions of 64% (*IL-6*) and 68% (*IL-8*), respectively (Fig. 6A & B).

No concentration dependency was observed, but solubility of the tablets was an issue at the higher concentrations in neutral medium. Therefore, incomplete dissolution of the tablet material likely affected and limited the effectively available concentration of *Opuntia* extract.

## Discussion

In the present study we characterized the activity profile of a recently developed new heartburn self-medication therapy (Refluthin^®^) based on a combination of mineral salt- based antacids (CaCO_3_ & MgCO_3_) with a polysaccharide-rich extract containing protective substances from *Opuntia ficus-indica* cladodes. In an artificial stomach model with constant addition of hydrochloric acid, we demonstrated a buffer capacity of approximately 9 mmol HCl per tablet (Fig. 1) and a rapid onset of action with an increase of artificial stomach fluid to pH 5.3 within 3 min after addition of one tablet in artificial saliva (Fig. 2). The buffer activity was mediated exclusively by the antacid components in the combination product (450 mg calcium carbonate plus 50 mg magnesium carbonate) and not affected by addition of the *Opuntia* extract (75 mg) (Fig. 1). The pH neutralization kinetics within less than 3 min suggests a fast onset of action concerning stomach acid neutralization and thus presumably a rapid relief of symptoms in patients. The maximal pH achieved (pH 5.4) in our study guarantees a mild pH increase, without exceeding into basic pH values. Our data on buffering capacity of the combination product is comparable to results of a recently published study with an antacid tablet containing a slightly higher concentration of 680 mg calcium carbonate and 80 mg magnesium carbonate^16^. In summary, our data suggest that intake of a single antacids-*Opuntia* extract combination product tablet should lead to a significant albeit transient improvement of excessive stomach acidity within minutes which is not negatively affected by combination with *Opuntia* extract.

In addition to the carbonate-based antacids, the combination product contains 75 mg of a dry polysaccharide-rich extract containing protective substances from *Opuntia ficus-indica* cladodes. Experimental gastritis models have shown gastroprotective effects of *Opuntia* based preparations^9,11^ and an Opuntia extract included in combination products exerted beneficial effects in patients with gastric discomfort^12,13^.

In vitro studies reported the formation of large colloidal aggregates of *Opuntia* mucilage-derived high-molecular weight polysaccharides in solution^14^. In addition, constituents of an *Opuntia* extract were shown to bind to CaCo-2 cells^15^, which suggests the ability to form viscous films on the surface of gastrointestinal epithelial cells.

We hypothesized that inclusion of an extract from *Opuntia ficus-indica* cladodes to the chewable tablet would complement the activity of the antacids by formation of a physically acting protective film on esophageal cells.

When we employed a low pH challenge to induce esophageal cell cytotoxicity, ground combination product tablets and the corresponding amount of carbonate antacids showed protective activity as would be anticipated based on the acid neutralization capacity, whereas the *Opuntia* extract was inactive (Fig. 3). We then developed a model of non-acidic reflux suitable to assess protective activity of treatments that was independent of the acid neutralization activity of the carbonates. A 30 min exposure to a high DCA concentration (1000 µM) proved to be suitable to induce measurable but not excessive esophageal cell irritation with concomitant induction of proinflammatory cytokines and a moderate level of cytotoxicity (Fig. 4). In this setting, pretreatment of cells with the *Opuntia* extract led to significant attenuation of inflammatory cytokine induction, IL-6 protein levels in supernatant and cytotoxicity (Fig. 5). The maximal protective effect of the ground tablets (Fig. 6) was similar to the effect size produced by pure *Opuntia* extract (62–68% reduction in gene expression, Fig. 5). This suggests that in the DCA model the activity of the tablets was primarily mediated by the *Opuntia* extract.

To the best of our knowledge, this is the first demonstration of the cytoprotective activity of an *Opuntia ficus-indica* cladode extract against simulated reflux challenge in esophageal cells.

In our assays the protective activity of *Opuntia* extract was limited to a short-term (30 min) exposure to DCA. In contrast, no effect was observed in the low pH model with a 3 h exposure time to the challenge (Fig. 3) and when DCA was applied constantly for 2–8 h without wash-out (supplementary figure S2). The time-dependency of the activity in the DCA-assay argues against a pharmacological mechanism of action mediated by direct interaction with the cell, e.g. via inhibition of intracellular signaling pathways. The data rather suggest that *Opuntia* extract temporarily attenuates access of some noxious stimuli to the cell but does not create a completely impermeable barrier. Our data are compatible with the concept that *Opuntia* extract creates a physically acting barrier on the surface of esophageal cells that reduces the exposure to noxious stimuli.

There are some limitations to our study. With COLO-680 N we used a single cell line derived from an esophagus squamous cell carcinoma and we did not confirm the results with other cell lines or primary tissues. Prichard et al. previously demonstrated that DCA induced contrasting effects on cell expression of integrin in cells lines derived from esophageal squamous epithelium (HET-1α) vs. non-dysplastic metaplasia (CP-A)^17^. Differential effects on IL-8 induction by DCA for different cell lines depending on their origin were also reported by Quilty et al.^18^. However, the response of our COLO-680 N cells to DCA was qualitatively similar to HET-1α and OE33 cell lines where DCA also resulted in induction of IL-6 or IL-8^18,19^ and thus seems to be representative. Another limitation to the extrapolation of the results is the use of acidified medium and DCA for simulation of acidic and non-acidic reflux, respectively, and use of the read-outs of cytotoxicity and inflammation for reflux-induced cell irritation. Apart from hydrochloric acid that was employed in our cytoprotection assay, gastric juice additionally contains other components such as pepsin and mucins which may have an impact on cell irritation. However, pepsin activity is dependent on low pH and thus addition of carbonates would not only neutralize acidity of the medium but also simultaneously inactivate pepsin as well^16^. Hence, we expect that protection against acidified medium is predictive for protection against acidic gastric juice.

We employed DCA as a model challenge to simulate non-acidic reflux. The relevance of bile acid-mediated esophageal inflammation has been primarily discussed as a cause of pathological changes in reflux esophagitis leading to Barrett’s esophagus^20^, i.e. in a generally more severe patient population as compared to the typical user of symptomatic self-medication therapies against heartburn. This increases the complexity of extrapolation of the in vitro observed treatment effects to the clinical situation of use in heartburn patients. Another unknown aspect is whether the concentrations of *Opuntia* extract required for cell protection can be actually achieved in the clinical setting. We determined attenuation of proinflammatory gene induction to short-term DCA stimulation at *Opuntia* extract concentrations of ≥ 0.1 mg/mL (Fig. 5) and for tablet concentrations of ≥ 0.48 mg/mL (Fig. 6). Assuming a dilution of a combination product tablet in 7.5 mL of saliva during chewing, the maximal concentrations of the swallowed suspension that gets into contact with the esophagus is 10 mg/mL for *Opuntia* extract and 160 mg/mL for the tablet. Hence, the observed active concentrations could be plausibly reached even if further dilutions by saliva and wash-out are considered.

In conclusion, our in vitro data demonstrate rapid pH neutralization of artificial stomach juice and protection of esophageal cells against deleterious effects of simulated acidic and non-acidic reflux by an antacids-*Opuntia ficus-indica* extract combination product. In our models, the polysaccharide-rich extract containing protective substances from *Opuntia ficus-indica* cladodes confers additional protective effects against non-acidic reflux challenge, compatible with the concept of formation of a physically acting barrier, without compromising the well-established neutralization activity of the carbonate-based antacids.

## Materials and methods

### Test items

The commercial combination product used in this study (Refluthin^®^ chewable tablets mint flavored) was provided by Dr. Willmar Schwabe GmbH & Co. KG (Karlsruhe, Germany). The dry polysaccharide-rich extract containing protective substances from *Opuntia ficus-indica* cladodes (Opuntia extract, Opunxia^®^70) was purchased from Bionap S.r.l. (Belpasso, Italy).

CaCO_3_ was from Particle Dynamics (Seymour, USA) and MgCO_3_ was purchased from Carl Roth (Karlsruhe, Germany). The COLO-680 N cell line (CLS 300464) was from CLS Cell Lines Service GmbH (Eppelheim, Germany).

### Assessment of acid neutralization capacity

For measurement of the buffer capacity an artificial stomach juice was prepared according to the following protocol:

NaCl (1450 mg), KCl (350 mg), K_2_HPO_4_ (135 mg), pepsin 500 mg (Sigma-Aldrich), mucin 1500 mg (Sigma-Aldrich) were dissolved in 450 mL deionized water and adjusted to a pH of 2 with 10% HCl (m/m) and subsequently filled up to 500 mL with deionized water.

The following test conditions were prepared:

1. One chewable tablet ground to powder.
2. 450 mg CaCO_3_ + 50 mg MgCO_3_.
3. 450 mg CaCO_3_ + 50 mg MgCO_3_ + 75 mg Opuntia extract without the tablet excipients.
4. 75 mg Opuntia extract.

The pH measurements were performed on a Methrom 905 Titrando titration robot equipped with a Methrom 814 USB Sample Processor. For the measurements, the artificial stomach juice was maintained at 37 °C. Samples were dissolved in 40 mL artificial stomach juice each. A time resolved pH measurement was performed to assess neutralization capacity with a titration rate of 0.2 mmol HCl/min by addition of 10 µL 1 M HCl solution every 3 s. The measurements were continued until the pH fell below 1. Data points were recorded by Methrom tiamo software (v2.5) and subsequently exported and processed by Microsoft Excel (v365).

### Determination of pH neutralization kinetics

For the determination of the neutralization kinetics, artificial saliva was prepared according to the following protocol:

NaCl (200 mg), KCl (200 mg), MgCl_2_ (0.4 mg), CaCl_2_ (250 mg), Na_2_HPO_4_ × 2 H_2_O (390 mg), Na_2_S x 2 H_2_O (1.3 mg), Urea (500 mg), Mucin (2000 mg) (Sigma-Aldrich, Taufkirchen, Germany) were dissolved in 450 mL deionized water and adjusted to a pH of 6.83 with 0.5 M NaOH and subsequently filled up to 500 mL with deionized water. The pH measurements were performed on a WTW pH 597 pH-meter equipped with a SI Analytics Blue Line pH 14 electrode, which was calibrated with standard solutions of pH = 7.0 and pH = 4.0 before measurement. For the measurements, the artificial stomach juice was maintained at 37 °C and stirred continuously by a magnetic stirrer. One chewable tablet was ground to powder and suspended in 5 mL artificial saliva. The slurry was added by a plastic Pasteur pipette to 100 mL of artificial stomach juice. To simulate gastric acid secretion, 0.1 M HCl solution was continuously added by an Ismatec MCP peristaltic pump equipped with a Masterflex L/S Easy-Load model 07516-00 pump head calibrated for a flow rate of 3 mL/min. This resulted in an acid secretion rate of 0.3 mmol H^+^/min. To simulate gastric emptying, a constant volume of 1.5 mL/min was removed from the gastric juice compartment by another peristaltic pump (Fig. S1, see supplementary figures). Time-resolved pH measurements were continued until the pH fell below 1. The study was performed in triplicates. Data points were recorded by time lapse photography of the pH meter with one data point every 15 s for the first 5 min, followed by one data point every 5 min for one hour. The data points were subsequently transferred manually to Microsoft Excel (v365) and analysis was carried out by GrapPad Prism v9.3.0.

### Low pH cytoprotection assay

#### Cell culture

COLO-680 N cells were grown in 175 mL flasks in cell culture medium (Minimum Essential Medium Eagle, Sigma M5650 + 10% FCS + 1% non-essential amino acids + 1% penicillin/ streptamycin + 2 mM L-glutamine). Cells were harvested 2–3 days before the cytoprotection study and seeded into 96 well plates with a density of 15.000 cells / well.

#### Test items

Solutions or suspensions of ground chewable tablets, Opuntia extract and carbonates (CaCO_3_ & MgCO_3_) were prepared in cell culture medium at the desired test concentrations.

### Cytoprotection assay

The culture medium of the 96 well cell culture plates was removed by aspiration and 50 µL of medium (RPMI 1640 without FCS & pen/strep) or test item solutions (ground tablets, corresponding amount of *Opuntia* extract or corresponding amount of CaCO_3_ & MgCO_3_, respectively) prepared in cell culture medium at 2-fold of the intended final concentration were added.

After 30 min of pre-incubation with test items (37 °C, 5% CO_2_), acid-mediated cytotoxicity was induced by addition of 50 µL of RMPI 1640 medium previously adjusted to pH 1.6 with HCl. For the 100% viability control wells, medium at pH 7.4 was added instead.

Set-up studies showed that the pH in the medium containing cells adjusted to 3.0 by this protocol in the absence of test items. This acidification was shown to be sufficient to induce a significant reduction in cell viability in the absence of test items.

After 3 h of incubation at 37 °C and 5% CO_2_, 100 µL of CellTiter Glo 2.0 reagent (Promega, Walldorf, Germany) was added to each well for the assessment of ATP levels as a read-out for the number of viable cells. The plates were shaken for 2 min to facilitate lysis of cells. After centrifugation at 200 *g* for 5 min, 150 µL of the supernatant were transferred with multichannel pipettes into white opaque plates (Costar) for assessment of luminescence signals in a SpectraMax M2 (Molecular Devices, San Jose, USA) cell plate reader (all wave lengths, 30 reads/well) after a stabilization period of 10 min.

Set-up studies demonstrated the linearity of the ATP signal within this cell number range and the different pH conditions and hence relative luminescence units (RLU) were used for assessment of cell viability without transformation to ATP concentration with a standard curve.

### Bile acid-induced cell irritation assay

#### Cell culture

COLO-680 N cells were cultured as described above. Cells were harvested 3–4 days before the bile acid-induced inflammation study and seeded into 48-well cell culture plates with a density of 100.000 (-3 days) or 75.000 cells (-4 days) / well, respectively.

#### Test items

Deoxycholic acid (DCA) was purchased from Sigma-Aldrich (≥ 98% HPLC purity). Stock solutions of 100 and 200 mM were prepared in ethanol and diluted in cell culture medium to a working solution in 1% ethanol.

*Opuntia* extract and ground tablets were dissolved in cell culture medium at the desired test concentrations.

#### Concentration- and time-dependency of DCA-induced gene induction

The culture medium of the 48-well cell culture plates was removed by aspiration. Then, 250 µL of either medium (RPMI with FCS & pen/strep + 1% ethanol) or DCA in cell culture medium at the intended final concentration (500 or 1000 µM in 1% ethanol) were added at different time points to achieve different exposure times (at 37 °C in incubator) with DCA for gene induction. Exposure times of 15 min, 30 min, 60 min, 90 min and 120 min (DCA) or 120 min (medium controls) were used. Following the exposure to DCA, the cells were washed twice with PBS to remove DCA. Then, cell culture medium was added and cells were incubated for 4 h to allow for a sufficiently high degree of gene induction prior to RNA isolation.

#### Assessment of effects of Opuntia extract and antacid-Opuntia combination product on DCA-induced inflammatory gene induction

The culture medium of the 48-well cell culture plates was removed by aspiration. Then, 100 µL of either medium (RPMI with FCS & pen/strep) or test item solutions prepared in cell culture medium at the intended final concentrations were added. After an incubation time of 30 min at 37 °C in the incubator to allow for film-formation of *Opuntia* extract on the surface of the cells, 100 µL of 2000 µM DCA in medium + test item + 1% ethanol was added as a challenge to induce inflammatory gene induction. Based on the previous kinetic study, an incubation time with DCA for 30 min was used. Then, cells were washed with PBS to remove both test items and DCA and cells were incubated in medium for 4 h prior to isolation of RNA.

#### Reverse transcription quantitative PCR

For total RNA isolation the ReliaPrep RNA Cell Miniprep System (Promega, Walldorf, Germany) was used. At the end of the 4 h-incubation period, cells were carefully washed twice with PBS (control by microscopy to prevent loss of cells). Then, 250 µL of lysis buffer (from isolation kit) was added and lysates were transferred to 1.5 mL reaction tubes and stored at -80 °C. After thawing of the reaction tubes at room temperature, 85 µL of isopropanol was added and mixed by vortexing for 5 s. Then, the purification protocol from the manufacturer´s instructions was followed. Finally, the elution tubes containing the purified RNA were stored at -70 °C.

Cellular RNA was quantified by RT-qPCR using Bio Rad kits:

- RT-qPCR: iScript Reverse Transcription Supermix for RT-qPCR (Bio Rad, Feldkirchen, Germany, Cat.No #1708841).
- qPCR: SsoAdvanced Universal SYBR Green Supermix (Bio Rad Cat.No. 1725274).

The following primers were used:

##### Target genes

Prime PCR Assay, Bio Rad, 20x.

- Interleukin 6 (IL-6), human Prime PCR Assay, Bio Rad Unique Assay ID qHsaCID0020314.
- Interleukin 8 (IL-8), human Prime PCR Assay, Bio Rad Unique Assay ID qHsaCED0046633.

##### Reference genes

Prime PCR Assay, Bio Rad.

- RPS 18 Prime PCR Assay, Bio Rad Unique Assay ID qHsaCEP0040177.
- HPRT1 Prime PCR Assay, Bio Rad Unique Assay ID qHsaCIP0030549.
- ACTB Prime PCR Assay, Bio Rad Unique Assay ID qHsaCED0036269.

Gene expression of IL-6 and IL-8 was quantified by the ΔΔ Ct method vs. the mean of the reference genes and expressed as fold-change vs. the mean of the corresponding medium controls in each plate.

#### Assessment of effects of Opuntia ficus-indica extract and antacids-Opuntia combination product on DCA-induced cytotoxicity and IL-6 release

COLO-680 N cells were pretreated with *Opuntia* extract and Refluthin^®^ tablets for 30 min, followed by a 30 min challenge with DCA as described for the gene induction assay. After washing, cells were incubated for 24 h at 37 °C. Then, cells were centrifuged (1000 g, 15 min, 4 °C) and 100 µL of supernatant was collected and stored at -80 °C prior to quantification of IL-6 protein by Bio Plex (Pro Human Cytokine Screening, Bio Rad, Feldkirchen, Germany) according to the manufacturer´s protocol. Cell viability was assessed by analysis of ATP levels in lysed cells using the CellTiter Glo 2.0 reagent (Promega) as described for the low pH cytoprotection assay. As relative luminescence units (RLU) at the elevated cell concentrations used in this assay were not linear, the actual ATP concentration was determined by use of an ATP standard curve. IL-6 protein concentration was normalized to ATP content in each well to counteract interference by treatment effects on viability and thus number of IL-6 producing cells.

### Statistical analysis

The pH time course in the acid neutralization kinetics is expressed as means ± SD of three independent assays. Time course data of DCA-induced gene induction from a single experiment with *n* = 3 technical replicates are shown as means ± SD.

For the cell protection and gene induction intervention studies, 3–5 independent studies were conducted with technical replicates of *n* = 3 (DCA assay) and *n* = 6 (low pH assay) per condition. Individual assay mean values and combined means ± SEM are shown in graphs.

Statistical significance was determined by one-way ANOVA (time course of DCA-induced gene induction) and two-way ANOVA (intervention studies), followed by Dunnett`s post-test vs. control treatment with stressor (low pH or DCA, respectively). *P* < 0.05 was considered statistically significant. Statistical analysis was conducted with GraphPad Prism version 9.3.0 for Windows, GraphPad Software, San Diego, California USA.

## Electronic supplementary material

Below is the link to the electronic supplementary material.

Supplementary Material 1

## Data Availability

The datasets generated during and/or analysed during the current study are available from the corresponding author on reasonable request.

## Abbreviations

ACTB: Beta actin
ATP: Adenosine triphosphate
DCA: Deoxycholic acid
FCS: Fetal calf serum
GERD: Gastroesophageal reflux disease
HCl: Hydrochloric acid
HPRT1: Hypoxanthin-phosphoribosyl-transferase 1
IL-6/IL-8: Interleukin-6/-8
PBS: Phosphate buffered saline
PPI: Proton pump inhibitors
RLU: Relative luminescence units
RPS18: Gene for 40 S ribosomal protein S18
