# Supplementary material for: A combination of carbonates and Opuntia ficus-indica extract protects esophageal cells against simulated acidic and non-acidic reflux in vitro
Source: Sci Rep. 2024 Sep 27;14:22355. doi: 10.1038/s41598-024-74047-7 (PMC11437097; doi:10.1038/s41598-024-74047-7)
Supplement: Supplementary file 1 — Supplementary Material 1 [file 41598_2024_74047_MOESM1_ESM.pdf]

A combination of carbonates and Opuntia ficus-indica extract protects esophageal cells against simulated acidic and non-acidic reflux in vitro

Supplementary Figure S1

Martin D. Lehner*, Ulrike Scheyhing, Jens Elsässer

Preclinical R&D, Dr. Willmar Schwabe GmbH & Co. KG, Karlsruhe, Germany

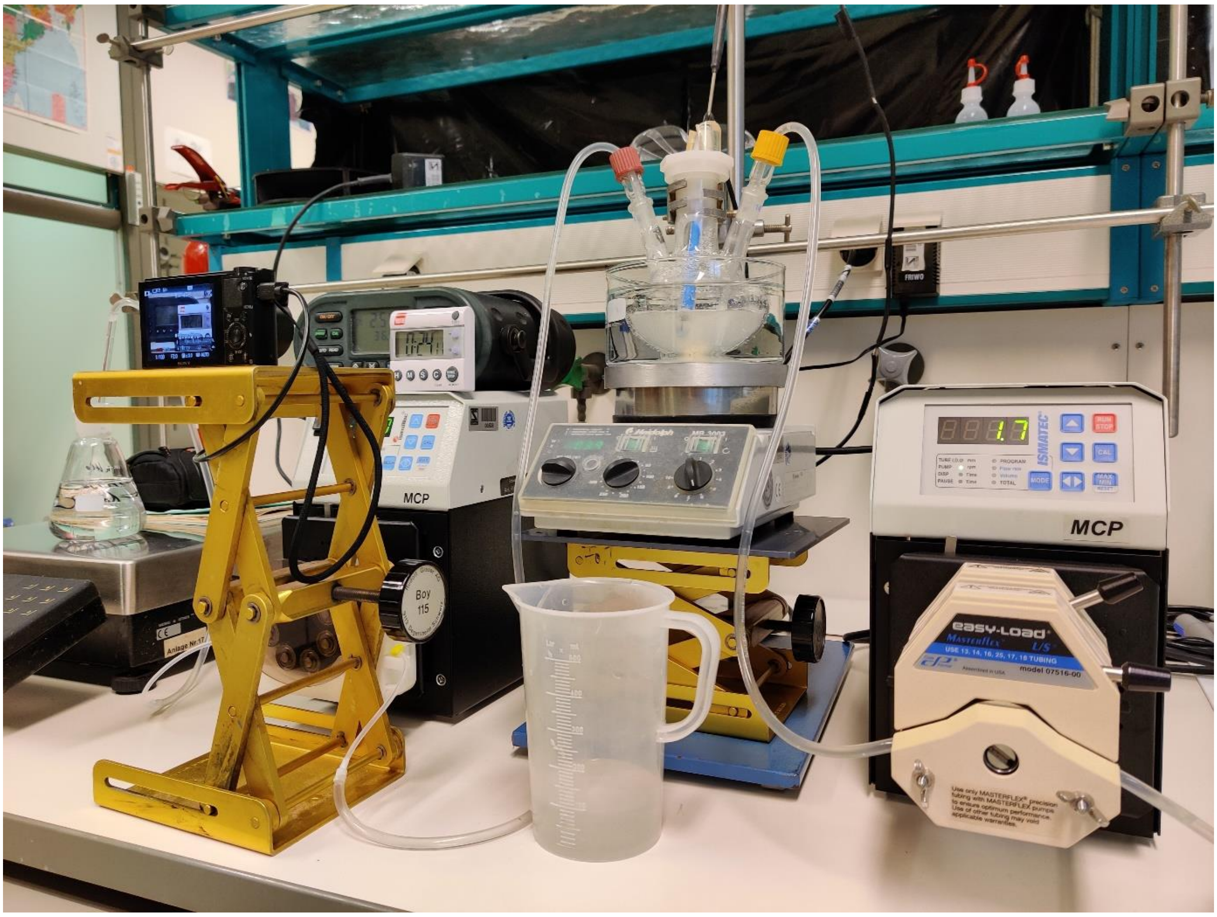

Supplementary Figure S1
Experimental setup for the determination of the neutralization kinetics.
From left to right: Camera for time lapse photography of the pH values on a platform. pH-meter and stopwatch on top of the peristaltic pump adding the HCl solution. Three-neck round-bottom flask as artificial stomach, tempered and stirred by the magnetic stirrer below. The pH electrode is dipped in the solution through the middle neck, while adding and draining of the solution is facilitated through the left and right neck, respectively. Additional peristaltic pump on the right for draining the solution.

A combination of carbonates and Opuntia ficus-indica extract protects esophageal cells against simulated acidic and non-acidic reflux in vitro

Martin D. Lehner*, Ulrike Scheyhing, Jens Elsässer, Žarko Kulić

Preclinical R&D, Dr. Willmar Schwabe GmbH & Co. KG, Karlsruhe, Germany

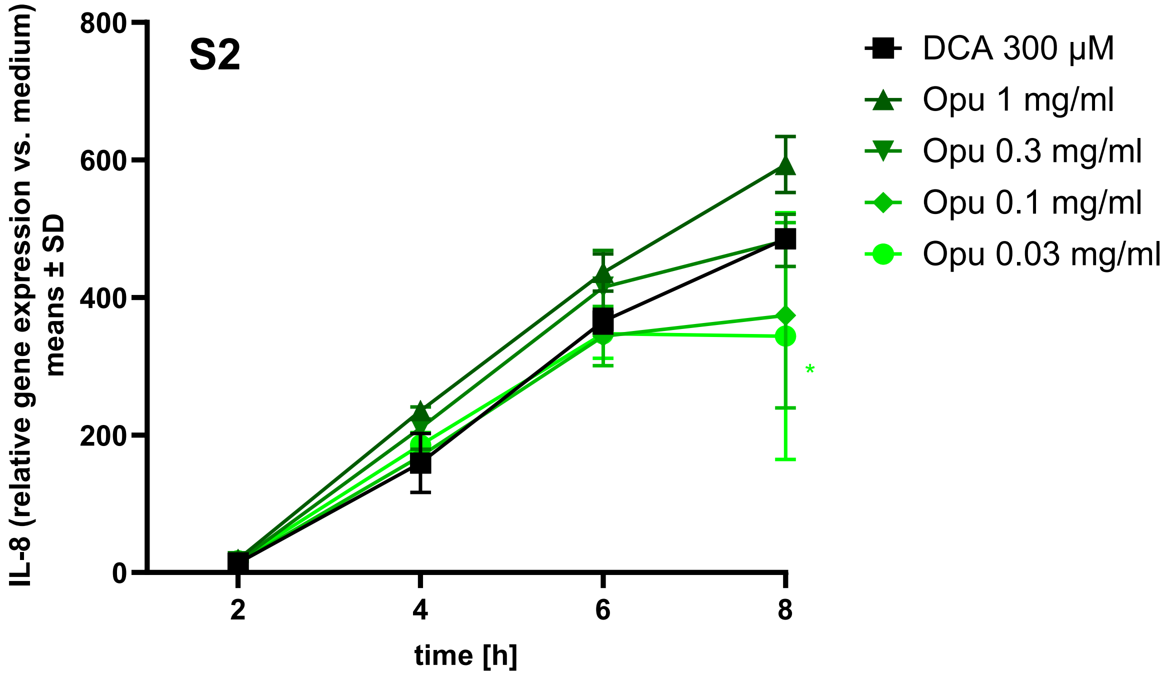

Supplementary Figure S2: ***Opuntia extract does not attenuate DCA induced IL-8 mRNA induction upon continuous prolonged exposure to DCA***

*Pretreatment of Colo 680N with Opuntia extract prior to constant exposure to DCA 300 µM for 2h-8h without wash-out did not consistently attenuate the DCA induced increase in IL-8 mRNA. Means ± SD of 3 technical replicates * p < 0.05 assessed by Two-Way ANOVA, followed by Dunnett´s test vs. DCA.*
